# Reduced guanidinoacetate in plasma of patients with autosomal dominant Fanconi syndrome due to heterozygous P341L *GATM* variant and study of organoids towards treatment

**DOI:** 10.1002/jmd2.12442

**Published:** 2024-08-19

**Authors:** Ignacio Portales‐Castillo, Rhea Singal, Anastasia Ambrose, Jong Hee Song, Minsoo Son, Young Ah. Goo, Wen Zhou, Avram Z. Traum, Ariella Coler‐Reilly, Benjamin D. Humphreys, Roberto Civitelli, Harald Jüppner, Andrew L. Lundquist, Peter Seres, Andrew S. Allegretti, Saadet Mercimek‐Andrews

**Affiliations:** ^1^ Department of Medicine, Division of Nephrology Washington University in St. Louis St. Louis Missouri USA; ^2^ Bone and Mineral Division Washington University in St. Louis St. Louis Missouri USA; ^3^ Department of Medical Genetics Faculty of Medicine and Dentistry University of Alberta Edmonton Alberta Canada; ^4^ Mass Spectrometry Technology Access Center at the McDonnell Genome Institute Washington University School of Medicine St. Louis Missouri USA; ^5^ Endocrine Unit Massachusetts General Hospital, and Harvard Medical School Boston Massachusetts USA; ^6^ Division of Nephrology Boston Children's Hospital, Harvard Medical School Boston Massachusetts USA; ^7^ Pediatric Nephrology MassGeneral for Children, Harvard Medical School Boston Massachusetts USA; ^8^ Division of Nephrology Massachusetts General Hospital, Harvard Medical School Boston Massachusetts USA; ^9^ Department of Radiology and Diagnostic Imaging, Faculty of Medicine and Dentistry University of Alberta Edmonton Alberta Canada

**Keywords:** arginine‐glycine amidinotransferase (AGAT), chronic kidney disease, creatine, Fanconi syndrome, *GATM*, guanidinoacetate, kidney organoids

## Abstract

Autosomal dominant Fanconi syndrome due to a *GATM* variant (GATM‐FS), causes accumulation of misfolded arginine‐glycine amidinotransferase (AGAT) in proximal renal tubules leading to cellular injury. GATM‐FS presents during childhood and progresses to end‐stage kidney disease (ESKD) in adults. We study creatine metabolism in two individuals of unrelated families with a known *GATM* variant and the effect of creatine supplementation in kidney organoids. Plasma and urine metabolites were measured by mass spectrometry. Brain creatine was assessed by magnetic resonance spectroscopy (MRS). Guanidinoacetate (GAA) synthesis by the AGAT mutant was measured in patient‐derived immortalized lymphocytes using stable isotopes of arginine and glycine. The effect of creatine on *GATM* expression was assessed in human kidney cells and organoids. Several family members from two unrelated families were diagnosed with Fanconi syndrome and had the c.1022C>T (p. P341L) variant in *GATM*. Two affected individuals in both families had moderately reduced plasma GAA levels. In comparison to wild‐type cells, GAA synthesis by patient‐derived *GATM* ^P341L+/−^ lymphoblastoid cell lines (LCL) was reduced, but not absent as in *GATM* cells from a patient with creatine deficiency syndrome. In vitro studies on human kidney organoids revealed reduced AGAT expression after treatment with creatine. Finally, we showed in one patient that creatine supplementation (5 g daily) substantially increased plasma creatine levels. We report low plasma and urine GAA in patients with autosomal dominant GATM‐FS and show that creatine downregulates AGAT in human kidney cells.

**Synopsis**

We study creatine metabolism in patients with Fanconi syndrome due to heterozygous *GATM* variants and show downregulation of AGAT in kidney organoids.

## 1 INTRODUCTION

Renal Fanconi syndrome is characterized by a defect in the reabsorption of bicarbonate, amino acids and glucose in the proximal convoluted tubules (PT) that leads to biochemical abnormalities, including metabolic acidosis, hypokalemia and excessive urinary losses of glucose, amino acids, and phosphate.^1^ Individuals with Fanconi syndrome can have a wide range of clinical features including anorexia, vomiting, failure to thrive, short stature, rickets, osteomalacia, low bone mass, and muscle weakness.^2^

Autosomal dominant renal Fanconi syndrome linked to heterozygous *GATM* (GATM‐FS) variants was first described by Reichold et al.,^1^ and subsequently reported in five additional families.^2^, ^3^, ^4^, ^5^, ^6^ The *GATM* gene located on chromosome 15q21.1 encodes arginine‐glycine amidinotransferase (AGAT), a 48 kDa mitochondrial enzyme that is expressed in various tissues, including the proximal renal tubules (PT), where it catalyzes the formation of guanidinoacetate (GAA), the precursor of creatine. Biallelic pathogenic *GATM* variants result in AGAT deficiency, which causes global developmental delay, cognitive dysfunction, intellectual disability, and muscle weakness.^7^ Affected individuals have markedly decreased or absent GAA and creatine levels in different body fluids, and absent or markedly decreased brain creatine in brain magnetic resonance spectroscopy (MRS). Notably, early creatine supplementation improves neurodevelopmental outcomes.^8^, ^9^, ^10^, ^11^, ^12^

The mechanism of disease in GATM‐FS is ascribed to the accumulation of AGAT mutant proteins in the PT, most likely the consequence of protein misfolding, resulting in tubular damage.^1^ Affected individuals present with renal Fanconi syndrome during childhood and can progress to end‐stage kidney disease (ESKD) during adulthood, presumably from chronic PT injury.^1^, ^4^, ^5^

Here, we report two unrelated, newly diagnosed families with autosomal dominant GATM‐FS, and study creatine synthesis, as well as the potential benefits of creatine supplementation in one patient. We also show functional defects of the mutant AGAT in vitro and demonstrate *GATM* expression and regulation in human kidney organoids.

## 2 MATERIALS AND METHODS

### 2.1 Individuals with autosomal dominant *GATM* ‐related Fanconi syndrome

The proband in family 1 was identified and evaluated at the Massachusetts General Hospital (Boston, MA). The proband and available family members provided written consent to participate in the study (MGH‐IRB: 2001P001063). The patient in family 2 were referred to S.M‐A in Canada. Targeted exome sequence analysis (Natera) of genomic DNA from the index case in family 1 revealed the previously reported c.1022C>T variant in *GATM*, which was confirmed by Sanger sequencing in our laboratory using forward primer 5′‐CACAGGGACGGCTAAGTTGTA‐3′ and reverse primer 5′‐GTCCCAAGCACCACTTCACA‐3′. Cycler protocol: initial denaturing at 95°C for 2 min, followed by 35 cycles of denaturing at 95°C for 30 s, annealing 62°C for 30 s, and 72°C for 60 s; final extension 5 min. Genomic DNA from 10 members of family 1 was then analyzed using the same primers and amplification conditions. The patient in family 2 underwent targeted next generation sequencing panel for renal tubular acidosis and Fanconi syndrome, as described in results section.

### 2.2 Metabolite quantification by targeted mass spectrometry

For Family 1, metabolites were extracted from plasma (100 μL) and urine (50 μL) by adding the 80% (v/v) methanol at a volume ratio of 10:1 then kept at −80°C during overnight. The samples were centrifuged at 20 000×*g* for 30 min at 4°C, supernatant was transferred to new vials and dried by SpeedVac with no heat. The dried samples were reconstituted with 50 μL of HPLC‐grade water and spiked with an equal volume of heavy isotopes of each analyte (Final concentration, 100 ng/mL) for internal standard. Peak area ratio (PAR) was calculated by light standard (unlabeled) peak area versus heavy standard (Isotope‐labeled) peak area.

Metabolites were analyzed in LC–MS/MS system using Vanquish™ Horizon UHPLC (Thermo Scientific) coupled with Orbitrap Exploris™ 240 (Thermo Scientific). Waters™ Acquity HSS T3 C18 (2.1 × 150 mm, 1.8 μm) was used for 10 min gradient separation at a flow rate of 400 μL/min with mobile phase A (0.1% Formic acid in Water) and mobile phase B (90% Acetonitrile with 0.1% Formic acid). Analyses were carried out using a Heated Electrospray Ionization (HESI‐II) probe in positive mode. For the parallel reaction monitoring (PRM) of target compound, fragment ions were scanned using Orbitrap at 15 000 resolution, and collision energy was optimized by stepwise increment. Skyline 23.1 was used for PRM data processing for quantification.

The calibration curve was generated by serial dilution of unlabelled standards with an equal amount of spiked in isotope‐labeled internal standards. Each calibration point was analyzed in triplicates. The concentration of metabolite was calculated by 1/*x* weighted linear regression using PAR (Light to heavy) and the LOD was taken as the *y*‐intercept for calculation.

The light standards of Creatine (C0780), Creatinine (C4255), and GAA (G11608) were obtained from Sigma‐Aldrich (MO, USA). The isotope‐labeled heavy standards of Creatine‐d3 (D‐1972), and Creatinine‐d3 (D‐3689) were obtained from CDN Isotopes (Quebec, Canada), and [1,2‐13C, 3‐15N] GAA(CNLM‐8300‐PK) was purchased from Cambridge Isotope Laboratory (MA, USA).

For one affected individual in family 2, plasma GAA and creatine levels were measured in a clinical biochemical genetic laboratory.

### 2.3 Enzyme assay

Lymphoblastoid cell lines (LCL cells) were generated at the Massachusetts General Hospital Mission Driven Service Core, as described previously,^13^ using whole blood from two patients of family 1 and one unaffected control without a *GATM* mutation. A *GATM*‐negative control from a patient with creatine deficiency syndrome was obtained from the Coriell Institute (Cat# GM27955). Enzyme activity assay was performed as previously described.^14^ Briefly, 100 μg of LCL protein lysates were incubated at 37°C with equal amounts of l‐[guanido‐15N2] arginine and [U‐13C2,15N] glycine at 10:16 ratios, for 20 h at 37°C. The reaction was stopped with perchloric acid at 70% and neutralized with KOH 6 mol. Metabolites were extracted as described above. The dried samples were reconstituted with 30 μL of water, and an equal amount of [13]C2 GAA was spiked into each sample (1 μg/mL) as an internal standard. The samples were analyzed on the same LC–MS/MS setting above.

The PAR of [1,2‐13C2/15N3] GAA relative to [13 C2] GAA was obtained for each sample, and single‐point calibration was performed by PAR multiplied by the known spiked amount of internal standard within the limit of quantification (LOQ) range from reverse calibration curve generated by serial dilution of [13 C2] GAA in metabolite extraction matrix.

Stable isotopes of l‐[guanido‐15N2] arginine (Cat# 204633‐92) and [U‐13C2,15N] glycine (Cat# 211057‐02‐2) were obtained from MedChemExpress [1,2‐13C2]. [13 C2] GAA was obtained from Santa Cruz (Cat# 634616‐40‐3; Purity, 98%).

### 2.4 Cell culture, RNA sequencing and analysis

Renal proximal tubular cells (HK‐2 cells), obtained from ATCC (Cat# 2190), were cultured in 6‐well plates using 2 mLs of Keratinocyte Serum Free Medium (K‐SFM) (Cat# 17005‐042) containing 5% FBS. After cells reached approximately 70% confluency, cultures were continued with the same medium or with medium containing creatine (final concentration: 5 mM; dilution of 10 μL in 1 mL). For RNA silencing, siRNA oligonucleotides (siGENOME) were obtained from Dharmacon, and cells were transfected according to the manufacturer protocol before adding creatine to the media. After 48 h of incubation with creatine, RNA was extracted with Direct‐zol RNA (Cat# R2050) and qRT‐PCR to quantify *GATM* expression was done using Origene SYBR Master Mix (Cat# QP10002), normalizing for β‐actin expression.

Primers used for at‐PCR: *GATM* forward 5′‐GATAGTGGGCAGAGCAGAAA‐3′, reverse 5′‐TTTACCAGAAGCAAGGAGGG‐3′. *βactin* forward 5′‐GGATCAGCAAGCAGGAGTATG‐3′ and reverse 5′‐AGAAAGGGTGTAACGCAACTAA‐3′.

### 2.5 Organoid differentiation and creatine treatment

Kidney organoids were generated following the Takasato protocol^15^ using BJFF6 induced pluripotent stem cells (iPSC), obtained from Benjam Humphreys Lab and the Pediatric Centers of Excellence in Nephrology (PCEN) at Washington University in St. Louis. On day 24, the organoid medium was supplemented with equal volumes of 50 μL of water or creatine (final concentration: 10 mM) dissolved in water for 48 h before harvesting. The control media STEMdiff™ APEL™2 Medium (STEMCELL™ Cat:05275) used to differentiate organoids is based on animal product‐free medium (APEL),^16^ contains arginine (1 mM), glycine (1 mM), no creatine, and equal volumetric amounts of water as the organoids supplemented with creatine. The organoids were differentiated in 3 separate batches (biological replicates) of the same inducible pluripotent cell line (BJFF.6) using 2–3 organoids for each condition at each batch of differentiation (2–3 technical replicates for each repeat).

### 2.6 Immunofluorescence and Immunohistochemistry

Formalin‐fixed, paraffin‐embedded 5 μm sections were deparaffinized with xylene, rehydrated and treated with 0.3% hydrogen peroxide in methanol. Antigen retrieval was achieved by placing the samples in a pressure cooker and incubated for 3 min at full pressure in citrate buffer (10 mM citric acid, pH 6.0), followed by gradual cooling to room temperature (RT). Sections were then blocked using serum blocking solution (Invitrogen HISTOSTAIN‐SP KIT) and incubated with primary antibody GATM (1:100; Proteintech) in PBS/0.1% Triton™ X‐100 (Sigma‐Aldrich) for immunohistochemistry (IHC) or GATM antibody and biotinylated Lotus tetragonolobus lectin (LTL) (1:300) primary antibodies for immunofluorescence (IF) overnight at 4°C. For IF, following washes in PBS, sections were incubated in appropriate secondary antibodies (Alexa Fluor® 488 Anti Rabbit; Invitrogen, A‐21428) and Alexa Fluor® Streptavidin 488 (Invitrogen, S11223) for 60 min at room temperature. Following more washes, sections were mounted in Slowfade Gold Antifade Mountant with DAPI (Thermofisher, S36938). AGAT and LTL were visualized using Leica DMi8 inverted Microscope at ×400 magnification, and the images were processed using Leica Application Suite X (LAS X) software or Hamamatsu NanoZoomer 2.0‐HT System. Quantification of positive AGAT and LTL areas was done using Image J by splitting color channels, adjusting threshold in each channel, and analyzing area with the function of analyzing particles.

For IHC, after incubation with primary antibody, sections were washed 3 times with PBS and incubated at room temperature with biotinylated universal secondary antibody (Life Technologies). After washing with PBS 3 times, secondary antibodies were visualized using a Vectastain ABC kit (Vector Laboratories, PK‐4000) and ImmPACT DAB Substrate Kit, Peroxidase (HRP) (Vector Laboratories, SK‐4105). Sections were then counterstained using Gill II Hematoxylin followed by washing in PBS and dehydration in ascending ethanol series and xylene.

### 2.7 Magnetic resonance spectroscopy

3D T1‐weighted images and chemical shift image (CSI) multi‐voxel MRS were acquired on 3 Tesla Siemens Skyra (Erlangen, Germany) at the University of Alberta Hospital, Edmonton. T1‐weighted image (Magnetization Prepared Rapid Gradient Echo Imaging, MPRAGE sequence, sagittal plane, field of view [FoV] 250 × 250 × 176 mm, resolution 0.98 × 0.98 × 1 mm, repetition time [TR] = 2200 ms, echo time [TE] = 2.45 ms, inversion time [TI] = 900 ms, flip angle [FA] = 8 deg) were used for tissue segmentation (gray matter [GM]/white matter [WM]/cerebral spinal fluid [CSF]). CSI MRS (semi‐adiabatic localization by adiabatic selective refocusing (sLASER) sequence, axial‐oblique plane, resolution 6.25 × 6.25 × 10 mm, 8 × 8 voxel matrix, TR = 1520 ms, TE = 135 ms) was used to assess metabolites (creatine compound and choline compound) in basal ganglia and white matter (internal capsule) regions.

CSI MRS data were analyzed using FSL‐MRS^17^ first, T1‐weighted images were segmented to GM/WM/CSF using fsl_anat (FSL v 6.0.7.11),^18^ and this segmentation was provided as one of the inputs for FSL‐MRS fitting. Basis set for the CSI sequence was generated using MRI Cloud (https://braingps.mricloud.org/mrs-cloud).^19^, ^20^ Two regions of interest were defined as masks (in CSI space) covering left basal ganglia (caudate nucleus, putamen and globus pallidus) as well as left internal capsule using fsleyes and spectra fitting was done for each of the two regions. Based on the report generated by FSL‐MRS fitting, we report a ratio of creatine compound (Creatine + Phosphocreatine, Cr + PCr) over choline compound (Glycerophosphocholine + Choline‐containing Compounds, GPC + PCh).

### 2.8 Literature review

The PubMed database was searched using *GATM* and Fanconi Syndrome key words. All references were reviewed for additional case reports in the published articles. American College of Medical Genetics and Genomics and the Association for Molecular Pathology (ACMG/AMP) variant classification guidelines for interpretation of variants were applied.^21^ All variants in the Genome Aggregation Database (gnomAD v3.2.1) (http://gnomad.broadinstitute.org/about) for their allele frequency in the general population were searched.

## 3 RESULTS

### 3.1 Family 1

The female index patient was first diagnosed with renal tubular acidosis and nephrolithiasis at age 17 (serum bicarbonate 19 mmol/L). At the time, she also had stage 2 CKD with a creatinine of 1.2 mg/dL and an estimated glomerular filtration rate (eGFR) of ~60 mL/min. She was started on hydrochlorothiazide for stone prevention and developed mild hypokalemia (3.3 mEq/L), thus treatment with this medication was stopped. Ten years later, she transitioned to the adult nephrology clinic for follow up. She had no further episodes of nephrolithiasis. Her creatinine remained at ~1.2 mg/dL (Supplemental Table 1). Her urine chemistry revealed glucosuria (495 mg/dL) with normal serum glucose level and pH of 7. She had marked generalized amino aciduria, phosphaturia (FePO4 of 27%) and calciuria (300 mg/24 h). Her 1,25(OH)2 vitamin D and parathormone levels were normal. Her family history was remarkable for a younger sister with a diagnosis of Fanconi syndrome, and her father with a diagnosis of ESKD at the age of 30 years, who received a renal transplant shortly after diagnosis.

**FIGURE 1:**
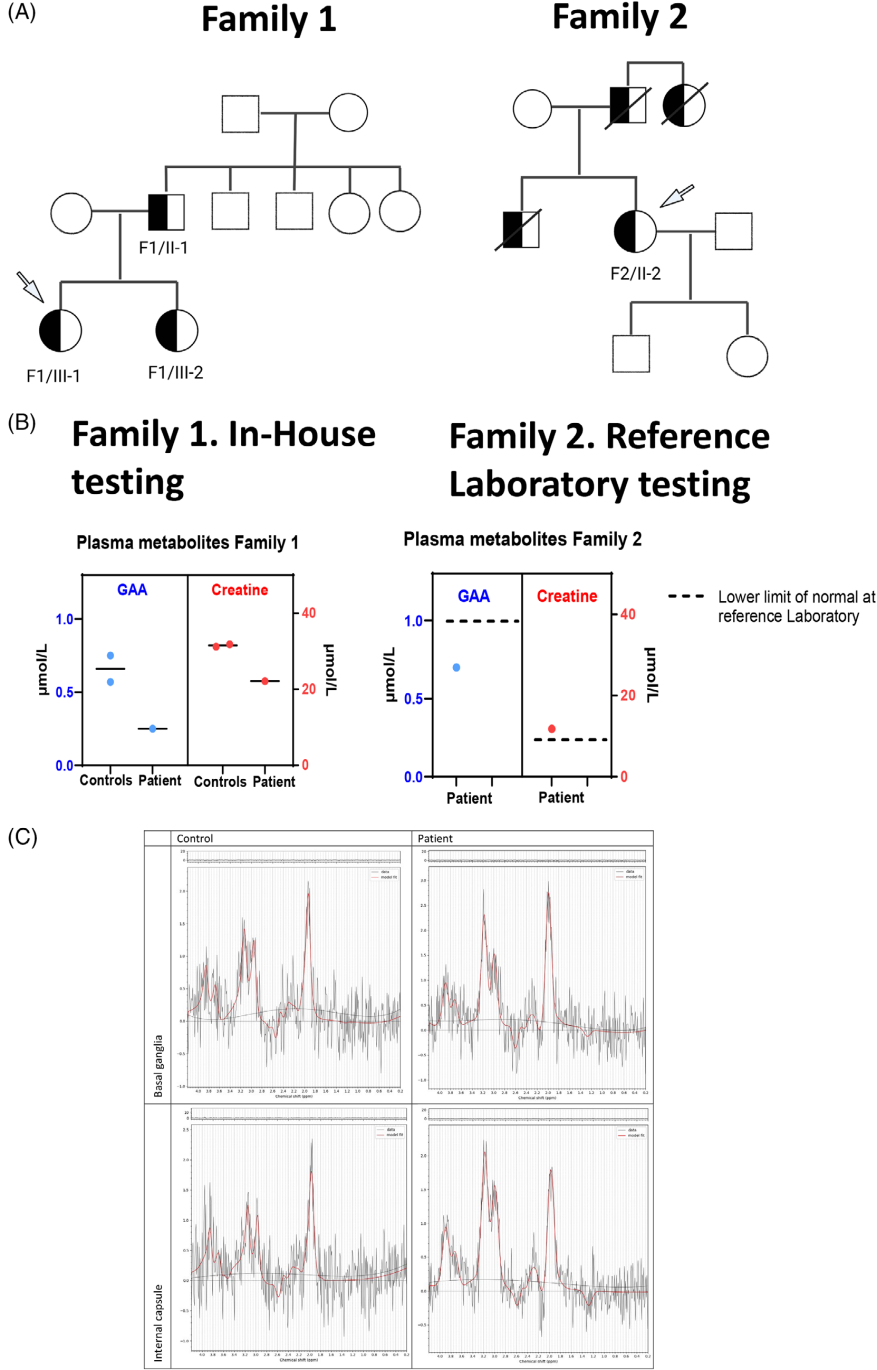
Creatine metabolites in families with CKD and *GATM* variants. (A) Pedigree of families 1 and 2. A *GATM* (c.1022C>T) variant was identified in the proband of Family 1 by commercial gene testing. Subsequently, we obtained DNA from blood and performed Sanger sequencing of *GATM* in all available family members. Only 3/10 were positive for *GATM P341L* heterozygous variant. The three of them had clinical CKD, while none of the affected relatives has CKD. In family 2, four first‐degree relatives were affected by CKD but only the proband was tested and found positive for the variant (/ = deceased). (B) The plasma and urine metabolites were measured in the proband and 2 healthy female relatives that donated blood from family 1. The measurements for family 1 (“In‐House testing”) were done by MTAC core at Washington University and repeated at the Massachusetts General Hospital, for a total of three repeats with comparable results, using plasma of healthy relatives as controls The graphic results are from a representative measurement. The metabolites in the patient in family 2 were measured at a reference laboratory with reference range illustrated in the graphic. The levels of plasma guanidinoacetate (GAA) in the patients in family 1 and 2 were 0.25 and 0.7 μmol/L, respectively. The levels of creatine in the two patients were 22.1 and 11.8 μmol/L, respectively. The laboratory reference range used to test the proband in family 2 were 1.1–3.3 μmol/L for GAA and 7.1–96 μmol/L for creatine. The lower limit of the laboratory reference range is marked with a discontinuous line in family 2. family. Published reference ranges for adult females, which can used to compare our results for patient in Family 1 are 0.87–3.15, 1.2–3.6 and 1–3.5 μmol/L for plasma GAA and 12.8–96.8, 12–99, 6–50 μmol/L for plasma creatine.^22^ (C) Brain magnetic resonance spectroscopy (chemical shift imaging, sLASER with TE = 135 ms). Fitted spectra are shown for control (70 year old female, left column) and individual with GATM‐FS (66 year old female, right column) for two regions—deep gray matter (basal ganglia, top row) and white matter (internal capsule, bottom row).

The index patient underwent targeted exome sequencing for CKD by commercial testing (Natera), using a gene panel including 385 genes associated with CKD. Testing revealed a known heterozygous variant (c.1022C>T; p. P341L) in *GATM*. We then obtained DNA from her parents, sister and all available second‐degree relatives including paternal grandparents, for Sanger sequencing of *GATM* gene. Her sister and father revealed the same *GATM* variant, but the variant was not identified in any other family member, including her father's siblings and the paternal grandparents. Thus, the variant had most likely occurred de novo in her father, who then transmitted the genetic defect to his two daughters (Figure 1A).

To test the metabolic consequences of *GATM* P341L variant, we measured the levels of GAA, creatine and creatinine in the plasma and urine of the patient using Mass Spectrometry at the Washington University of St. Louis and the Massachusetts General Hospital. Levels of plasma GAA were approximately 2‐fold lower in this patient compared to two non‐affected female relatives 0.25 versus 0.75 and 0.57 μmol/L and mildly lower for plasma creatine 22.1 versus 31.8 and 31.2 μmol/L (Figure 1B). While the levels of plasma GAA in the patient were >3‐fold lower than published reference ranges (0.87–3.15, 1.2–3.6 and 1–3.5 μmol/L),^22^ the plasma creatine was within the low range of normal for female adults ~13–20 μmol/L.^22^, ^23^ In contrast, serum creatinine in the patient was elevated at 88.8 versus 53.3 and 50.9 μmol/L in the two controls. The urine GAA/creatinine ratio in the patient was 21.8 ± 0.49 versus 241.2 and 65.3 mmol/mol of two female relatives and 87.6 and 38.7 mmol/mol of two male relatives. Urine creatine/creatinine ratio was 98.6 mmol/mol creatinine, lower than two female controls (369.3 and 251.9 mmol/mol creatinine). We note that although lower than her unaffected relatives, used as controls, using our methodology, the urine GAA and creatine of the patient were within the lower limit of normal by reference standards.^22^

### 3.2 Family 2

This female index case had the diagnosis of autosomal dominant GATM‐FS at the age of 66 years after being referred to the medical genetics clinic to investigate underlying genetic causes of Fanconi syndrome progressing to ESKD requiring renal replacement therapy. She completed Bachelor of education and worked as a teacher. Chronic kidney disease (CKD) was found incidentally at the age of 30 years during insurance investigations. She had history of short stature, and hypothyroidism in her 40s, cataract and pulmonary alveolar proteinosis in her 60s. Her family history was remarkable for Fanconi syndrome and kidney failure requiring peritoneal dialysis in her father (died in his 60s), brother (died in his 50s) and Fanconi syndrome in her paternal aunt (died in her 60s). She has two healthy adult children with no history of Fanconi syndrome or CKD in their 30s (Figure 1).

Her urine analysis revealed proteinuria (1 g/24 h), glucosuria (2+) and generalized amino aciduria. Her kidney ultrasound showed diffuse increased echogenicity and markedly atrophic kidneys at the age of 64 years. Her plasma amino acid analysis, uric acid, lactate, and ammonia levels were normal. She underwent targeted next generation sequencing panel for renal tubular acidosis for five genes (*ATP6VOA4, ATP6V1B1, CA2, SLC4A1, SLAC4A4*) and 21 additional genes were added which were known to cause Fanconi syndrome (*ALDOB, ATP7B, BCS1L, CLCN5, CTNS, EHHADH, FAH, G6PC, GALT, GATM, HNF1A, HNF4A, LRP2, NDUFAF6, OCRL, SLC2A2, SLC34A1, SLC3A1, SLC7A7, SLC7A9, VPS33B*). A heterozygous known variant (c.1022C>T; p. P341L)^1^ in *GATM* was identified (Supplemental Table 2).

After the genetic diagnosis of GATM‐FS, we measured creatine metabolism biomarkers in a clinical biochemical laboratory in the United States. She had low GAA (0.7 μmol/L; reference range 1.1–3.3) and low normal creatine (11.8 μmol/L; reference range 7.1–96.5) in plasma. In the urine, she also had low GAA (0.7 mmol/mol creatinine; reference range 4–83) and low normal creatine (10.1 mmol/mol creatinine; reference range 3–178). Brain MRS revealed decreased creatine in basal ganglia and in white matter compared to age‐appropriate control who was a 70‐year old female with chronic progressive ophthalmoplegia, onset at age 66 years, and normal cognitive functions at the time of brain MRS. She has multiple mitochondrial deletions ranging in size between 8.6 and 13.1 kb in muscle specimen. An estimate rate of the heteroplasmy of all deletions together is 10%. The (Cr + PCr)/(GPC + PCh) ratio was 67% of age‐appropriate control in basal ganglia. The (Cr + PCr)/(GPC + PCh) ratio was 50% of age‐appropriate normal control in white matter (Figure 1C).

## 4 AGAT P341L ENZYME ACTIVITY

**FIGURE 2:**
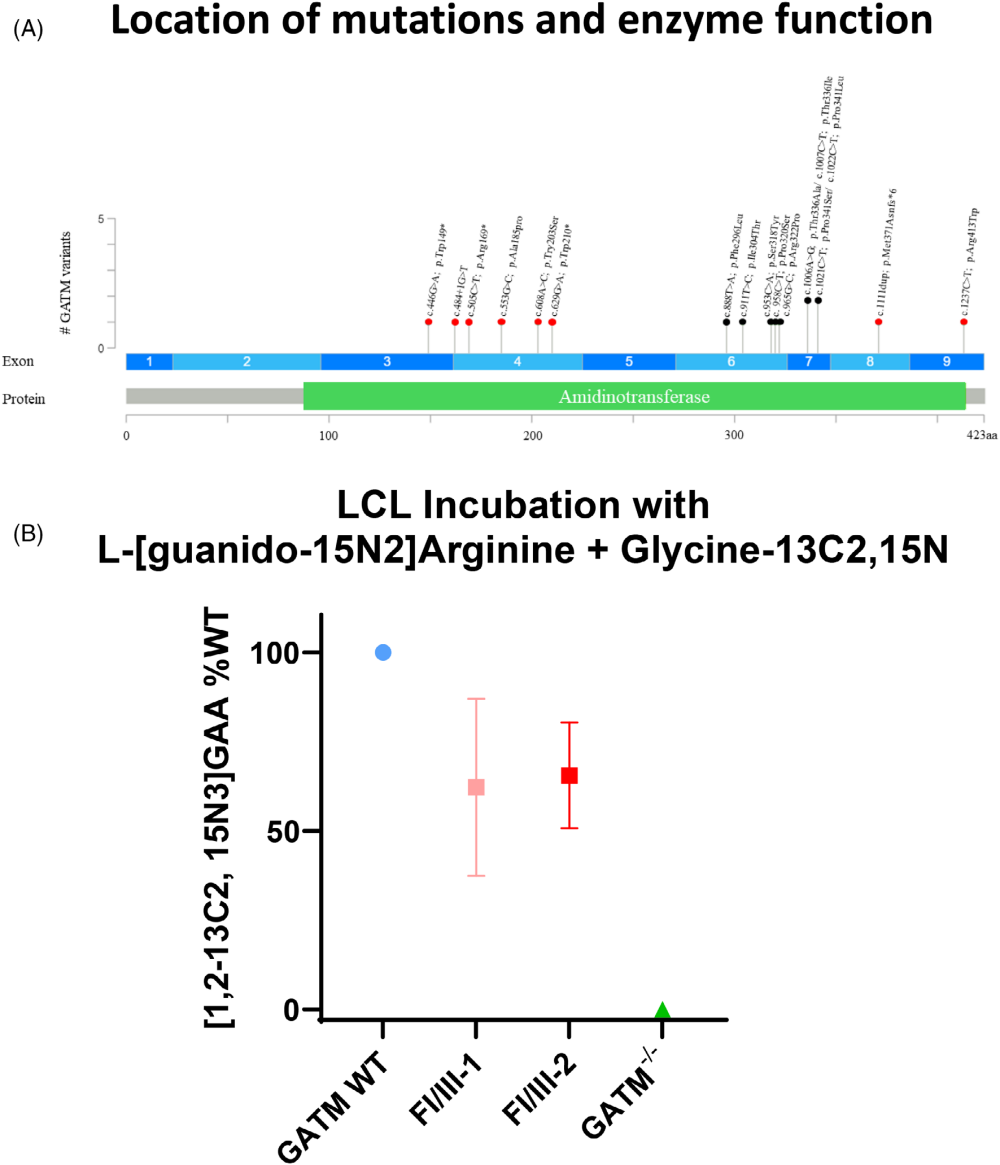
AGAT P341L enzyme activity. (A) Lollipop chart of variants in *GATM* and their phenotypes, including the amidinotransferase functional domain and visualized exons, red icons indicate variants causing AGAT deficiency, and black icons indicate variants causing autosomal dominant *GATM* related Fanconi syndrome. (B) Synthesis of GAA was measured in vitro using immortalized lymphocytes of two patients in family 1 (FI/III‐1 and FI/III‐2) and controls without *GATM* variant (WT) or cells from a patient with homozygous *GATM* variants causing creatine deficiency syndrome (*GATM* ^*−/−*^). The results showed lower GAA synthesis in two affected family members of family 1 compared to a control without *GATM* variant. The graphic results are normalized to the formation of GAA by cells obtained from a control without variants in *GATM* (100%). The experiment was repeated three times using separate cell vials each time, with 1–2 technical replicates in each measurement, with comparable results each time. In one repeat, the formation of GAA was quantified in nmol/hour/mg protein as 51.7 for the WT, 29.5 for FI/III‐1, 39.35 for FI/III‐2 and 0 for *GATM* ^*−/−*^.

Assessment of metabolite measurement in these two subjects with GATM‐FS suggested that the mutant AGAT P341L enzyme is partially dysfunctional. To directly corroborate this observation, we generated lymphoblastoid cells (LCL) from the two affected sisters in family 1 and obtained LCLs from an unrelated control without *GATM* variants (wild‐type *GATM*). We also obtained cells from a patient with homozygous *GATM* mutations associated with creatine deficiency syndrome (Coriell # GM27955) (*GATM* ^*−/−*^). We cultured LCLs in parallel and obtained protein lysates for incubation with stable isotopes of arginine and glycine, as previously described.^14^ Synthesis of GAA was detected in the control cells, and no GAA in cells obtained from the cells of the patient with creatine deficiency syndrome. LCLs derived from the two individuals with heterozygous *GATM* P341L showed that GAA production was reduced by 25%–50% relative to the positive control (Figure 2B). Item et al.^7^ measured GAA synthesis in the parents of patients with creatine deficiency syndrome and found it to be reduced by about 50%. Therefore, our data suggests that the mutant AGAT P341L has residual enzyme activity in vitro.

## 5 EFFECT OF CREATINE SUPPLEMENTATION IN A PATIENT WITH FANCONI SYNDROME

Based on previous report,^1^ the two affected sisters in family 1 were recommended to start 5 g/day creatine supplementation, a dose used for athletes and generally safe long‐term in healthy individuals.^24^ Much higher doses (400 mg/kg/day) are used for patients with AGAT deficiency.^11^ In one of our patients in whom we measured metabolites on and off creatine supplementation, creatine supplementation in the morning raised plasma creatine from 22 to 577 μmol/L approximately 5 h after the last ingestion of the supplement, while plasma GAA remained suppressed. The patient has tolerated creatine supplementation for more than 1 year and eGFR has remained stable. On her most recent testing, while in the second trimester of pregnancy, her serum creatinine and Cystatin C levels, as measures of renal function, have decreased to 0.85 mg/dL and 0.87 mg/L, respectively, possibly due to pregnancy‐related hyperfiltration, while urine amino acids excretion appears to have stabilized, as evidenced by a trend towards decrease in the amount of urine amino acids (Supplemental Table 3).

## 6 CREATINE DOWNREGULATES *GATM* IN HUMAN CELLS

**FIGURE 3:**
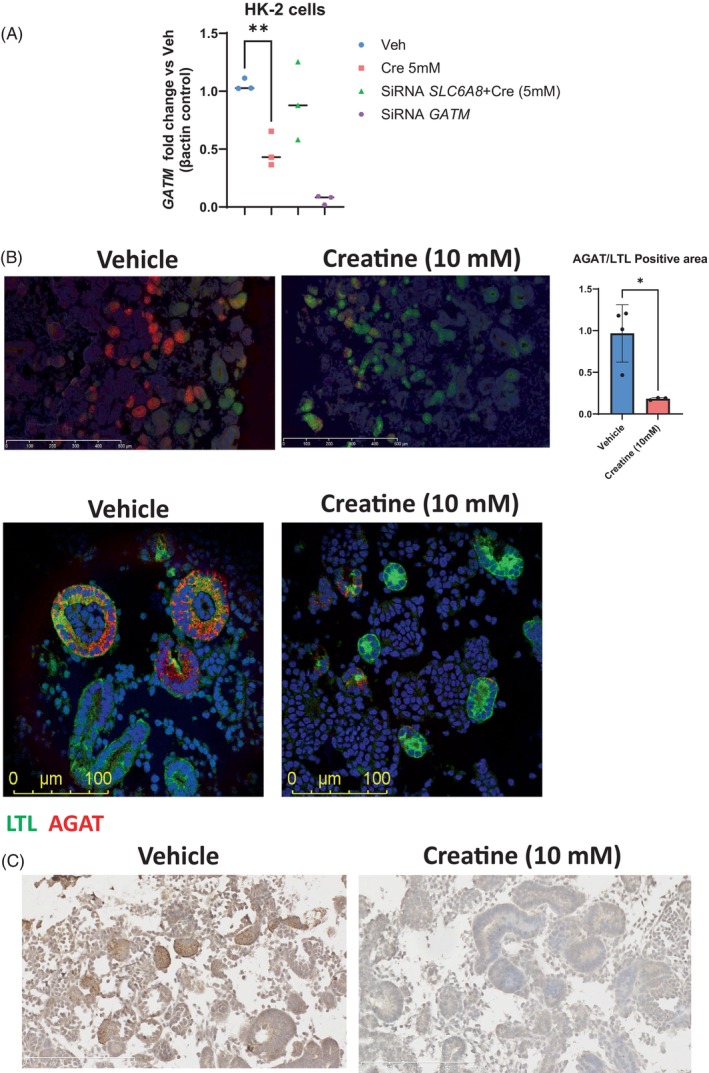
Creatine downregulates *GATM* expression in human cells. (A) Creatine downregulates *GATM* expression in PT cell lines (HK‐2 cells). The effect of creatine at 5 mM is eliminated by silencing of the creatine transporter SLC6A8. Figure is mean ± SD of 3 biological replicates, each done in triplicate. A two‐tail *T* test was used to establish significance and *p* values <0.05* were considered statistically significant. (B) Top panel: Representative images of kidney organoids are presented, using immunofluorescence (IF) methodologies to detect AGAT. For this experiment 3–4 separate vials of inducible pluripotent cells (iPSC) of the same cell line (BJFF6), were used to differentiate kidney organoids. After differentiation, 4 kidney organoids were supplemented with creatine in the media for 48 h and 3 were supplemented with equal volumetric amounts of water until collection. Entire organoids sections were imaged using Hamamatsu NanoZoomer. For quantification, areas of the same size as in the displayed IF figure were utilized to quantify positive areas for l‐Arginine:glycine amidinotransferase (AGAT) and Lotus tetragonolobus lectin (LTL) using Image J. The area of AGAT relative to LTL was compared among vehicle or creatine treated organoids. A two‐tail *T* test was used to compare and *p* values <0.05 were considered significant. Bottom panel: Confocal microscopy image of kidney organoid. We observed downregulation of AGAT in red by creatine in the PT, identified with the specific marker LTL in green. The figure is representative of 3–4 kidney organoids treated with either creatine or vehicle and differentiated in 3 separate biologic experiments. (C) Representative kidney organoid images using immunohistochemistry methodologies to detect AGAT. Entire organoids sections were imaged using Hamamatsu NanoZoomer.

Consistent with prior studies in wild‐type rodents, chickens and immortalized human cells,^25^, ^26^, ^27^, ^28^ we found that creatine supplementation downregulates *GATM* expression in human PT cell lines (HK‐2 cells). The effect of creatine on HK‐2 cells requires the presence of the creatine transporter (gene *SLC6A8*) (Figure 3A). To further study this effect of creatine, we generated kidney organoids from human induced pluripotent stem cells (iPSC) using the Takasato protocol.^15^ Starting on day 25, tubular structures appear and can be, identified as PT by the marker Lotus tetragonolobus lectin (LTL). Expression of AGAT was detected in PT developing in wild type kidney organoids exposed to vehicle; by contrast, AGAT abundance was substantially downregulated in organoids incubated with 10 mM creatine for 48 h (Figure 3B,C).

## 7 LITERATURE REVIEW

In total, 36 cases of genetically confirmed autosomal dominant GATM‐FS have been reported to date from 10 families.^1^, ^2^, ^3^, ^4^, ^5^, ^6^ In most individuals, symptom onset occurs in childhood,^1^, ^2^, ^4^ although two individuals from one family presented with osteomalacia at the ages of 42 and 62 years old,^3^ and one individual presented with glucosuria at age 34.^5^ Plasma creatinine was elevated during late adolescence or adulthood in almost all individuals.^1^, ^4^, ^5^ In approximately one third of individuals, there was documented progression to renal fibrosis and ESKD requiring renal replacement therapy between 30 and 70 years of age.^1^, ^4^, ^5^, ^6^ Hypophosphatemic rickets or osteomalacia have been reported in five individuals (13%).^2^, ^3^, ^4^ Common biochemical abnormalities include glucosuria (*n* = 35),^1^, ^2^, ^3^, ^4^, ^5^, ^6^ generalized aminoaciduria (*n* = 31),^1^, ^4^ and metabolic acidosis (*n* = 32).^1^, ^5^ So far, nine different missense variants in 10 unrelated families have been reported. ACMG/AMP variant classifications for all variants are summarized in Supplemental Table 2. All variants in *GATM* in individuals with AGAT deficiency and autosomal dominant *GATM* related Fanconi syndrome are shown in Figure 2A.^12^, ^13^, ^14^, ^15^, ^16^

In addition, we contacted Association for Creatine Deficiencies (ACD) (https://creatineinfo.org) to inquire about patients with heterozygous variant in AGAT to search for the possibility of undiagnosed Fanconi syndrome. Through ACD, we received information from a parent who has a child with AGAT deficiency due to homozygous c.484+1G>T *GATM* variant who was previously published in the medical literature.^29^ Both parents are carrier of this *GATM* variant, and their urine dipstick test was normal.

## 8 DISCUSSION

We report two new families with autosomal dominant GATM‐FS. In PT cells, *GATM* variants can form fibrillary aggregates in mitochondria, and similar aggregates have been observed in kidney biopsies, leading to the conclusion that accumulation of abnormal protein in PT may be the cause of CKD progression.^1^ However, the functionality of mutant AGAT in this disorder has not been studied. All known variants identified in individuals with autosomal dominant GATM‐FS cluster within a 45‐amino acid sequence in the amidinotransferase domain,^2^, ^3^, ^4^, ^5^ whereas the variants reported in individuals with AGAT deficiency are located throughout the entire amidinotransferase domain (Figure 2A).^1^, ^11^, ^12^, ^13^, ^14^, ^15^, ^16^ Here, we demonstrate that the mutant P341L AGAT enzyme is functionally impaired, resulting in moderately reduced GAA synthesis in vitro. Interestingly, low plasma GAA was present in affected members of both families; one individual in family 1 also had lower urine GAA compared to four unaffected relatives and the other individual in family 2 had low urine GAA as measured in a reference laboratory. The plasma and urine levels of creatine also were moderately reduced compared to controls (Family 1) or within the low normal range (Family 2).

The findings of low GAA in plasma and urine should be considered preliminary given the low number of samples and the different methodologies used. The moderate reduction in GAA and creatine in these patients does not appear to lead to any neurologic abnormality and it is unclear if it has any contribution to renal disease, but its plausible that there is at least some reduction in the normal negative feedback to *GATM* provided by creatine. Future studies are needed to compare the levels of plasma and urine GAA among patients with GATM‐FS and those with Fanconi syndrome from other causes and with carriers of variants associated with AGAT deficiency. Therefore, although creatine supplementation may not be necessary to treat a possibly affected creatine metabolism it would be very likely beneficial to treat the progressive CKD by downregulating the expression of AGAT and therefore reducing the abundance of pathological AGAT aggregates.

Interestingly, we report a new individual with GATM‐FS who had lower creatine in brain MRS compared to one age‐matched control. It was previously reported that one individual had normal creatine in gray and white matter in brain MRS.^1^ More brain MRS result will increase our understanding if individuals with autosomal dominant GATM‐FS have decreased creatine levels. Our data also supports investigating if low GAA levels can serve as a screening test for the identification of individuals with autosomal dominant GATM‐FS. Currently, searching for elevated levels of plasma GAA is part of neonatal screening in several states for early identification of patients with guanidinoacetate methyltransferase deficiency, since prompt treatment with creatine can prevent irreversible neurologic damage.^19^

Our report has several limitations including the low number of individuals with autosomal dominant GATM‐FS and the different methodologies (range references) utilized to measure the metabolites in the two unrelated families. It is also important to consider that the individual in family 2 had ESKD at the time of identification.

In conclusion, we report for the first time, low plasma GAA levels in individuals and a lower creatine in brain MRS compared to one age‐matched control in one individual with GATM‐FS as well as downregulation of AGAT in human kidney organoids. Our findings in human derived cells and organoids support the therapeutic potential of creatine to prevent AGAT accumulation in PT cells which may prevent CKD in adulthood.

## AUTHOR CONTRIBUTIONS

I.P.‐C., A.L.L., A.S.A., A.Z.T. and S.M.‐A. identified the patients and contributed to their diagnosis and care. I.P.C., R.S., A.A., P.S, W.Z., J.H.S., M.S., Y.A.G, A.C.‐R. generated the data. I.P.C., A.S.A, R.C., B.D.H., S.M.‐A., P.S. and H.J. interpreted the data. I.P.C. and S.M.‐A. were the lead writers of the manuscript. H.J., P.S., R.C. aided in the revision of the manuscript for content. All authors read and approved the manuscript. I.P.C. accepts full responsibility for the work and/or the conduct of the study, has access to the data, and controls the decision to publish.

## FUNDING INFORMATION

H.J. is supported by PO1‐DK11794, subproject 3 (NIDDK). The content of this manuscript has not been influenced by the sponsors.

## CONFLICT OF INTEREST STATEMENT

Ignacio Portales‐Castillo, Rhea Singal, Anastasia Ambrose, Jong Hee Song, Minsoo Son, Young Ah Goo, Wen Zhou, Avram Z. Traum, Ariella Coller‐Reilly, Benjamin D. Humphreys, Roberto Civitelli, Harald Jüppner, Andrew L. Lundquist, Peter Seres, Andrew S. Allegretti and Saadet Mercimek‐Andrews declare that they have no conflict of interest.

## ETHICS STATEMENT

This study was approved by MGH‐IRB: 2001P001063 and Washington University in St. Louis IRB: 202311182. For patient 2, there was no additional ethic approval required.

## PATIENT CONSENT

All procedures followed were in accordance with the ethical standards of the responsible committee on human experimentation (institutional and national) and with the Helsinki Declaration of 1975, as revised in 2000. Informed consent was obtained from all patients to be included in the study.

## ANIMAL RIGHTS

This article does not contain any studies with animal subjects performed by the any of the authors.

## Supporting information


**Supplemental Table 1.** The results of general chemistry laboratory are presented for patients in the two families. *Patient in Family 2 had End‐stage kidney disease at the time of diagnosis. 1. The reference range for plasma guanidinoacetate and creatine shown was obtained from the clinical laboratory that measured the values in the patient of family. The measurements of family 1 were done at two research facilities. Published reference ranges in female adult plasma are 0.87–3.15, 1.2–3.6 and 1–3 for plasma guanidinoacetate and 12.8–96.8, 12–99, 6–50 μmol/L for plasma creatine.^22^
**Supplemental Table 2.** ACMG/AMP variant classifications for all variants including published are summarized.
**Supplemental Table 3.** Urine amino acid trend in a patient in Family 1.

## Data Availability

Data archiving is not mandated but data will be made available on reasonable request.
