# Supplementary material for: Reduced guanidinoacetate in plasma of patients with autosomal dominant Fanconi syndrome due to heterozygous P341L GATM variant and study of organoids towards treatment
Source: JIMD Rep. 2024 Aug 19;65(5):341–53. doi: 10.1002/jmd2.12442 (PMC11558468; doi:10.1002/jmd2.12442)
Supplement: Supplementary file 1 — Supplemental Table 1. The results of general chemistry laboratory are presented for patients in the two families. *Patient in Family 2 had End‐stage kidney disease at the time of diagnosis. 1. The reference range for plasma guanidinoacetate and creatine shown was obtained from the clinical laboratory that measured the values in the patient of family. The measurements of family 1 were done at two research facilities. Published reference ranges in female adult plasma are 0.87–3.15, 1.2–3.6 and 1–3 for plasma guanidinoacetate and 12.8–96.8, 12–99, 6–50 μmol/L for plasma creatine. 22 Supplemental Table 2. ACMG/AMP variant classifications for all variants including published are summarized. Supplemental Table 3. Urine amino acid trend in a patient in Family 1. [file JMD2-65-341-s001.docx]

**Supplemental Table 1:** Laboratory Results

| Variable | CASE 1 | CASE 2 |
| --- | --- | --- |
| ***Plasma*** |  |  |
| Sodium (mmol/L) (135-145) | 141 | 139 |
| Chloride (mmol/L) (97-110) | 104 | 109 |
| Potassium (mmol/L) (3.3-4.9) | 3.5 | 4.3 |
| Bicarbonate (mmol/L) (22-32) | 19 | 20 |
| Phosphorus (mg/dl) (2.3-4.5) | 2.2 | 3.4 |
| Creatinine (mg/dl) (0.6-1.1) | 1.2 | * |
| Guanidinoacetate (μmol/L) (1.1-3.3)^1^ | 0.25 | 0.7 |
| Creatine (μmol/L) (7.1-96.5)^1^ | 22.1 | 11.8 |

**Supplemental Table 2:** ACMG/AMP variant classifications

| **Gene NM#/Inheritance Pattern/Reference** | **Variant (Zygosity/Inheritance)** | **SIFT** | **MuTaster** | **Polyphen2 (HumVar)** | **Conservancy** | **gmAD allele count/allele #** | **Variant Type** | **Variant Classification** | **Chart classification** |
| --- | --- | --- | --- | --- | --- | --- | --- | --- | --- |
| *GATM* (NM_001482.3)/AD Reichold et al., | c.958C>T; p.Pro320Ser (Heterozygous) | Deleterious | Disease causing | Possibly damaging | 8/8 | Not found | Missense | Likely pathogenic | PM2, PP2, PP1, PP3, PP5 |
| *GATM* (NM_001482.3)/AD Reichold et al., | c.1006A>G; p.Thr336Ala (Heterozygous) | Deleterious | Disease causing | Benign | 8/8 | Not found | Missense | VUS | PM2, PP2, PP1 |
| *GATM* (NM_001482.3)/AD Reichold et al., | c.1007C>T, p.Thr336Ile (Heterozygous) | Deleterious | Disease causing | Possibly damaging | 8/8 | Not found | Missense | VUS | PM2, PP2, PP1 |
| *GATM* (NM_001482.3)/AD Seaby et al., | c.965G>C p.Arg322Pro (Heterozygous) | Deleterious | Disease causing | Probably damaging | 8/8 | Not found | Missense | Likely pathogenic | PM2, PP1, PP3, PP2, PP5 |
| *GATM* (NM_ NM_001482.2)/AD Kudo et al., | c.888T > A; p.Phe296Leu) (Heterozygous) | Deleterious | Disease causing | NA | 8/8 | Not found | Missense | VUS | PM2, PP4, PP1, PP2 |
| *GATM* (NM_ NM_001482.3)/AD Koyun et al., | c.953C>A; p.Ser318Tyr (Heterozygous) | Uncertain | Disease causing | NA | 8/8 | Not found | Missense | VUS | PM2, PP2 |
| *GATM* (NM_001482.3)/AD Reichold et al., and current study | c.1022C>T; p.Pro341Leu (Heterozygous) | Deleterious | Disease causing | Probably damaging | 8/8 | Not found | Missense | Likely pathogenic | PS3, PM2, PP1, PP3, PP5 |
| *GATM* (NM_001482.3)/AD Li et al. | c.911T>C, p.Ile304Thr (Heterozygous) | Deleterious | Disease causing | Probably damaging | 8/8 | 2/1613962 | Missense | VUS | PM2, PP3, PP2, PM6_supporting |
| *GATM* (NM_ NM_001482.3)/AD Ragate et al. | c.1021C>T, p.Pro341Ser (Heterozygous) | Deleterious | Disease causing | Probably damaging | 8/8 | Not found | Missense | Likely pathogenic | PP2, PM2, PM5, PP3 |

**Supplemental Table 3:** Urine amino acid trends

| **Component** | **Ref Range (nmol/mg Cr)** | **2010** | **2022** | **7/2023** | **2/2024** | **Change 2010 to 2022** | **Change 2022 to 7/2023** | **Change 7/2023 to 2/2024** |
| --- | --- | --- | --- | --- | --- | --- | --- | --- |
| Taurine | 24-1531 | 1297 | 767 | 438 | 944 | **Decrease** | **Decrease** | Increase |
| Asparagine | 25-238 | 591 | 1476 | 4715 | 2182 | Increase | Increase | **Decrease** |
| Serine | 97-540 | 986 | 2261 | 3336 | 2452 | Increase | Increase | **Decrease** |
| Glycine | 229-2989 | 3825 | 7660 | 7236 | 6855 | Increase | **Decrease** | **Decrease** |
| Glutamine | 93-686 | 3075 | 12028 | 9492 | 13281 | Increase | **Decrease** | Increase |
| Histidine | 81-1128 | 1513 | 2466 | 3113 | 2314 | Increase | Increase | **Decrease** |
| Threonine | 31-278 | 889 | 2840 | 5518 | 2865 | Increase | Increase | **Decrease** |
| Citrulline | <12 | 231 | 812 | 729 | 552 | Increase | Increase | **Decrease** |
| B-Alanine | <52 | 38 | 116 | 243 | 393 | Increase | Increase | Increase |
| Alanine | 56-518 | 986 | 4213 | 6373 | 4114 | Increase | Increase | **Decrease** |
| Glutamic Acid | <34 | 25 | 47 | 65 | 93 | Increase | Increase |  |
| 1-Methylhisitdine | 23-1339 | 1473 | 1859 | 716 | 1030 | Increase | **Decrease** | Increase |
| 3-Methylhistidine | 70-246 | 255 | 197 | 143 | 150 | **Decrease** | **Decrease** | Increase |
| Carnosine | <35 | 8 | 8 | 4 | 3 | None | **Decrease** | **Decrease** |
| Arginine | <114 | 122 | 723 | 1053 | 323 | Increase | Increase | **Decrease** |
| A-Aminoadipic | <47 | 45 | 42 | 90 | 72 | Increase | Increase | **Decrease** |
| B-Aminoisobutyr | <301 | 55 | 135 | 302 | 151 | Increase | Increase | **Decrease** |
| A-Amino-N-Butyr | <19 | 30 | 157 | 90 | 212 | Increase | **Decrease** | Increase |
| Proline | <26 | 589 | 2538 | 5162 | 2068 | Increase | Increase | **Decrease** |
| Ornithine | <25 | 111 | 496 | 1637 | 484 | Increase | Increase | **Decrease** |
| Cystathionine | <30 | 35 | 15 | 59 | <1 | Increase | Increase | **Decrease** |
| Cystine | 10-98 | 319 | 739 | 845 | 1017 | Increase | Increase | Increase |
| Lysine | 15-271 | 1061 | 2974 | 4734 | 3616 | Increase | Increase | **Decrease** |
| Methionine | <16 | 13 | 77 | 147 | 125 | Increase | Increase | **Decrease** |
| Valine | 11-61 | 234 | 1063 | 1750 | 2205 | Increase | Increase | Increase |
| Tyrosine | 15-115 | 192 | 626 | 717 | 864 | Increase | Increase | Increase |
| Isoleucin | <22 | 41 | 243 | 274 | 475 | Increase | Increase | Increase |
| Leucin | <51 | 102 | 366 | 620 | 763 | Increase | Increase | Increase |
| Phenylalanine | 13-70 | 136 | 481 | 611 | 948 | Increase | Increase | Increase |
